# Measuring continuing medical education conference impact and attendee experience: a scoping review

**DOI:** 10.5116/ijme.65cc.8c88

**Published:** 2024-02-29

**Authors:** Lisa Albrecht, Misty Pratt, Rhiannon Ng, Jeremy Olivier, Margaret Sampson, Neal Fahey, Jess Gibson, Anna-Theresa Lobos, Katie O'Hearn, Dennis Newhook, Stephanie Sutherland, Dayre McNally

**Affiliations:** 1 Children's Hospital of Eastern Ontario Research Institute, Ottawa, Canada; 2 ICES, Toronto, Canada; 3 University of Ottawa, Ottawa, Canada; 4 KPMG Canada, Quebec, Canada; 5 Dalhousie University, Halifax, Canada; 6 Children's Hospital of Eastern Ontario, Ottawa, Canada

**Keywords:** Medical education conference, conference evaluation, evaluation tool, conference scoping review, evaluation domain

## Abstract

**Objectives:**

The aim was to comprehensively identify published research evaluating continuing medical education conferences, to search for validated tools and perform a content analysis to identify the relevant domains for conference evaluation.

**Methods:**

We used scoping review methodology and searched MEDLINE® for relevant English or French literature published between 2008 and 2022 (last search June 3, 2022). Original research (including randomized controlled trials, non-randomized studies, cohort, mixed-methods, qualitative studies, and editorial pieces) where investigators described impact, experience, or motivations related to conference attendance were eligible. Citations were assessed in triplicate, and data extracted in duplicate.

**Results:**

Eighty-three studies were included, 69 (83%) of which were surveys or interview based, with the majority conducted at the end of or following conference conclusion. Of the 74 tools identified, only one was validated and was narrowly focused on a specific conference component. A total of 620 items were extracted and categorized into 4 a priori suggested domains (engagement-networking, education-learning, impact, scholarship), and an additional 4 identified through content analysis (value-satisfaction, logistics, equity-diversity-inclusivity, career influences). Time trends were evident, including the absence of items related to equity-diversity-inclusivity prior to 2019, and a focus on logistics, particularly technology and virtual conferences, since 2020.

**Conclusions:**

This study identified 8 major domains relevant for continuing medical education conference evaluation. This work is of immediate value to individuals and organizations seeking to either design or evaluate a conference and represents a critical step in the development of a standardized tool for conference evaluation.

## Introduction

Continuing medical education (CME) conferences are an integral part of health care. CME conferences are widely regarded as essential by clinicians, trainees, and the patients they serve as they support critical activities such as knowledge exchange, networking, and scholarly initiatives like research.^1^^-^^4^ The importance of CME conferences is further highlighted by their prominence in physician maintenance of certification^4^ and correlation between lack of opportunities to attend conferences and increased risk of burnout and feelings of inadequate knowledge or isolation.^5^  As an example, in a longitudinal study of emergency physicians opportunity to attend conferences was associated with a 3 times lower risk of burnout.^5^ Given the significance to health care and academia, there has been rapid growth and global expansion in the conference industry over the past century, with some estimates suggesting hundreds of thousands of events hosted globally each year.^6^^-^^8^ While this growth has benefits, it also presents significant downsides, including substantial time and financial investments (organizers and attendees) with increasingly recognized environmental consequences. A study of a single mid-sized American conference estimated that more than 10 000 tonnes of carbon dioxide were generated by air travel alone – equivalent to the annual amount produced by 550 US citizens.^9^ Moving forward, it is critical the field consider the costs and environmental impact of conferences, and strive to maximize value to attendees, patients, and the healthcare system.

Despite their importance and cost, there is no standardized means for conference evaluation, leading to several issues. First, with such a large number of conference options available within all specialities, attendees have no objective means of knowing which conferences provide the greatest value and/or best suit their individual needs.  Available evidence suggests the approach to conference design and implementation can significantly influence impact.  As an example, multiple studies show conferences that utilize both interactive and didactic seminars have more learning when compared to solely didactic or interactive seminars.^1^^, ^^10^^-^^13^ The lack of standardized evaluation tools makes it challenging for academic and industry researchers to demonstrate and quantify the value of new approaches, innovations and technologies. Consequently, conference organizers and their financers must make decisions about how to spend the limited resource (time and money) when designing their conference without access to this data.

To begin addressing the gap in high quality conference evaluation methodology, we sought to perform a scoping review of the published research evaluating CME conferences. Objective one was to comprehensively identify research studies evaluating conference experience, with the goal of identifying and examining the tools and frameworks utilized. Objective two was to compile a repository of frequently observed evaluation domains and subdomains based on information extracted from the studies. The findings of this scoping review will be of immediate use to individuals or organizations seeking to design or evaluate a conference and represents a critical first step in developing a standardized tool for conference evaluation.

## Methods

We prepared a scoping review protocol guided by established methodology^14^ and published the protocol on Open Science Framework 04-May-2021. The project was completed at a tertiary care pediatric hospital associated with the University of Ottawa (Ottawa, Canada).  Results are reported according to the PRISMA Scoping Review checklist (see supplemental digital appendix 1).

### Literature search and study selection

Two information specialists co-developed the search strategy using Peer-Review of Electronic Search Strategies (PRESS) Checklist principles^15^ in consultation with the review team, after identification of seven eligible (true positive) articles used for key word generation. Following information specialist advice (M.S.), we conducted the search solely in MEDLINE as it has indexing designed specifically to identify citations specific to conferences/congress. In databases without such indexing, it is difficult to selectively retrieve research about conferences (rather than conferences about research) due to the limitations of Boolean logic (see supplemental digital appendix 2).

We uploaded RIS files and screened citations using insightScope, a web-platform designed to facilitate a large-team or crowdsourcing approach to citation screening.^16^ Each citation was assessed independently and in triplicate at both the title-abstract and full-text screening levels (M.P., N.F., R.N., J.G., K.O., J.O., L.A.), with conflicts resolved by team consensus. Prior to title and abstract screening, a test set of 50 citations randomly selected from the full set (enriched with 5 true positives) were screened by all study team members to identify discrepancies and clarify eligibility criteria.^16^

### Inclusion criteria

We included English and French-language medical studies published from 2008 onward (last search conducted June 3, 2022). This date was chosen because the Medical Subject Heading term “Congresses as Topic” was introduced to the National Library of Medicine’s Resource Description Framework in the year 2008.^17^ We sought to identify studies representing original research where the investigators intended on evaluating, quantifying or describing impact, participant experience, or motivations for conference attendance. This included original research focused on the development or validation of an instrument (i.e., scale, score, instrument, survey, app) intended to evaluate conference impact or participant experience. A wide variety of study designs were eligible including randomized controlled trials, non-randomized studies, cohorts, mixed-methods, and qualitative studies.

Editorials, letters, commentaries, and opinion pieces were not eligible for inclusion unless the authors described the development of original research or creation of an evaluation tool or framework. Systematic reviews were to be retained to identify both potentially relevant studies from reference lists, and document conference outcomes of interest. To promote sensitivity, impact and experience were not rigidly defined, and screeners were encouraged to be inclusive. The populations of interest included conference organizers, attendees (health care professionals, trainees, and researchers), and other stakeholders (patients, caregivers, and policy makers). Studies were excluded if the conference was not related to health or medicine and if the format of the conference/congress was out of scope.

### Data collection and quality assessment

See supplemental digital appendix 3 for the full list of variables in data extraction. Data extraction was performed independently and in duplicate (M.P., N.F., R.N., J.G., K.O., J.O., L.A.), with disagreements resolved initially through consensus and then through consultation with the study lead (D.M.). The data extraction tool was developed using an iterative process by which study team members (D.M., M.P., N.F., R.N., AT.L.) participated in three rounds of data extraction for a total of 15 citations. A key component of data extraction was recording the individual outcomes and/or questions comprising the evaluation tools (e.g., surveys) included in the studies. When the tool was not provided, these items were extracted from the text, tables or figures in the article. Conference characteristics (e.g., attendance, location, timing of evaluation tool administration) were also extracted from article text. When available, we extracted variables related to the design of the evaluation tools, including any mention of validation studies, pilot testing, or use of methodological frameworks. Given the scoping nature of the review and expectation of significant heterogeneity (population, methodology), we did not a priori plan either meta-analyses or a formal assessment of the methodologic quality of the articles using a standardized tool.^18^ However, a general assessment of study quality using relevant elements common to quality assessment tools was performed (supplementary digital appendix 5).

### Analysis and statistics

Data related to study characteristics was reported descriptively using counts with percentages or measures of central tendency and variance (e.g., mean/median with SD/IQR). Results are presented descriptively in text, tables, and figures.

Content analysis was performed for domain and subdomain identification using deductive and inductive approaches^19^ (D.M., L.A., D.N., S.S.). For the deductive stage, we identified four a priori domains based on a preliminary literature review and team expertise: engagement/networking, education/learning, impact (patients and policy), and scholarship. During data extraction, two independent assessors identified items from each study, with each classified directly into one of the a priori domains, and the remaining items placed in an unassigned group. This approach was piloted on an initial set of 10 studies, and item extraction was then completed for the remaining studies by two independent assessors. The team inductively sorted unassigned items into four additional domains, and further content analysis was performed to organize items into subdomains where appropriate (see supplemental digital appendix 4 for additional details and example items in each category). All items were reviewed by study for identification of differences in item number, wording and classification, with conflicts resolved through consensus or involvement of another core team member. As this was a review, institutional ethical approval was not required.

## Results

### Search findings

**Figure 1:**
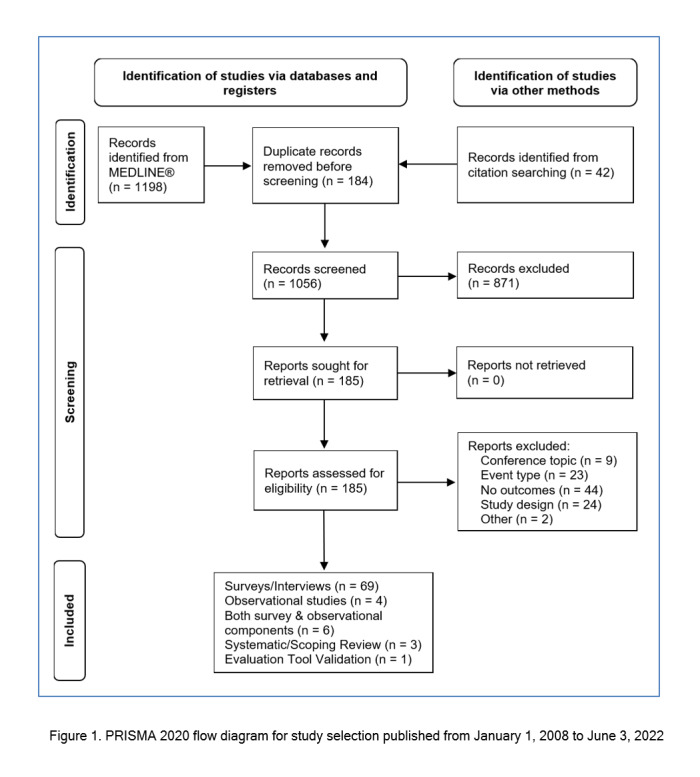
PRISMA 2020 flow diagram for study selection published from January 1, 2008 to June 3, 2022

The original search and updates identified 1198 citations. An additional 42 potentially relevant citations not retrieved from the search of MEDLINE were identified during a review of the references lists of included studies. Following title and abstract screening, 185 studies were included for full text review. Of these, 83 were deemed eligible for analysis and included 69 surveys/interviews, 4 observational studies, 6 studies with both survey and observational components, 3 systematic/scoping reviews, and 1 tool validation study. The study screening process is summarized in the PRISMA 2020^20^ flow diagram in Figure 1.

### Conference study characteristics

**Figure 2:**
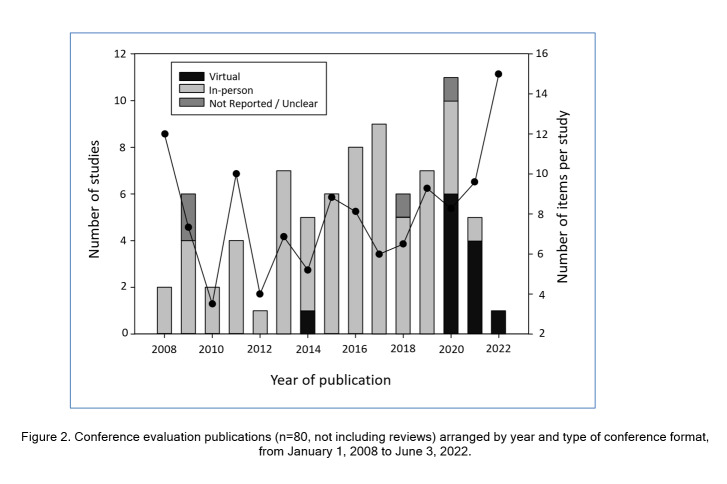
Conference evaluation publications (n=80, not including reviews) arranged by year and type of conference format, from January 1, 2008 to June 3, 2022.

Geographically, the majority of the studies were based in North America (n=60, 72%), with Europe representing the second largest locale (n=10, 12%). Topics of the conferences being studied spanned 25 fields of health care and special interest groups with radiology (n=10, 12%), health policy (n=6, 7%), and a surgical discipline (n=6, 7%) being the most prevalent. Figure 2 provides the number of studies by year of publication and demonstrates a gradual rise in publications between 2008 and 2013, followed by a plateau, and a spike in 2020.

While the minority of studies from 2008-2019 assessed virtual conferences (n=1, 2%) or those for which attendance method wasn’t specified (n=6, 9%), there was a clear shift from 2020 onwards with 11 (65%) studies assessing conferences hosted virtually. Additional characteristics of the 83 studies ^1^^, ^^3^^, ^^21^^-^^101^ are summarized in Table 1. Our assessment of study quality indicators demonstrated certain elements such as clarity of study objective(s) and design were well detailed in most publications (≥90%). However, other indicators such as approach to tool development (23%), adequate outcome description (30%) and participant response rates (57%) were often lacking (full details in supplementary digital appendix 5).

**Table 1:** Characteristics of included studies and the conferences they evaluated, published between January 1, 2008 to June 3, 2022

Conference Evaluation Studies (n=83)	
Origin of article, n (%)	
North America	60 (72.3)
Europe	10 (12.0)
Australia/New Zealand	4 (4.8)
East Asia	4 (4.8)
Africa	1 (1.2)
Central and South America	2 (2.4)
Not reported/unclear	2 (2.4)
Study Type, n (%)	
Survey/Interview	69 (83.1)
Observational studies	4 (4.8)
Both survey and observational	6 (7.2)
Systematic/Scoping review	3 (3.6)
Evaluation tool validation	1 (1.2)
Data Collection Methods, n (%)	
Quantitative	29 (34.9)
Qualitative	3 (3.6)
Mixed methods	45 (54.2)
NR/Unclear	3 (3.6)
NA*	3 (3.6)
Conference Specifics, n (%)	
Conference length reported^†^	51 (61.4)
Conference lengths unclear	5 (6.0)
Number of attendees reported^‡^	45 (54.2)
Number of attendees unclear	7 (4.8)
Participant recruitment, n (%)	
At conference	15 (18.1)
Electronically^⁋^	30 (36.1)
Both	8 (9.6)
Unclear/NR/NA	30 (36.1)
Survey/Interview measurement times^§^	
Before conference	21 (25.3)
At start of conference	1 (1.2)
During conference	8 (9.6)
End of conference	23 (27.7)
Post-conference	45 (54.2)
NR/NA	9 (10.8)
Conference evaluation method, n (%)^‖^	
Online	34 (41.0)
In person/at conference	12 (14.5)
Both	11 (13.3)
NR/Unclear	17 (20.5)

Abbreviations: Health care providers (HCP); Not applicable (NA); Not reported (NR) *Systematic or scoping reviews †Mean length was 2.6 days, ranged from 1 to 6 days ‡Number of attendees ranged from 43 to 18 000 ⁋In-person recruitment occurred at conferences; electronic recruitment methods included email, as part of online registration, or over social media. §30 studies evaluated conferences at multiple time points (only 3 of these provided the evaluation tool, and only 2 gathered related data before/after, so further analysis was not conducted). Some studies conducted measurements at multiple time points. Therefore, n (%) will be greater than the total number of included studies. ‖9 studies did not use evaluation tools.

### Characteristics of conference evaluation methodology

Of the 83 studies, 74 (89%) used evaluation tools that sought direct input (via surveys or interviews) from the conference attendees. The remaining 9 (11%) studies included systematic and scoping reviews, discussion based/open-forum reflections on the conference, and observational trials linking conference attendance to other metrics (example: exam performance).

Among the 69 (83%) studies providing data on study respondent number, the median was 99 (IQR: 50-220). While most studies (n=56, 68%) included trainees in their conference evaluation, relatively few considered patients and/or caregivers (n=7, 8%). The majority of studies performed evaluations either immediately at the end of the conference (n=23, 28%) and/or post-conference conclusion (n=45, 54%). The length of follow-up for the studies that measured post-conference evaluation was reported in 21 (25%) studies and ranged from 2 days to 5 years. Additionally, there were 22 (26%) studies that gathered data from conference participants before or at the onset of the conference and again at or post-conference conclusion. Table 1 provides additional details on the approach to conference evaluation and participant recruitment.

### Evaluation of tool quality and design

Of the 74 (89%) studies using surveys or interviews, 39 (53% of these studies) provided all or a portion of the tool. Of these, only one^97^ described their tool as having been validated and focused on participants’ attitudes related to a mobile device app intended for conference use. A second study reported using a partially validated tool,^77^ and specifically focused on how the conference impacted self-assessment of comfort with providing end of life care. For the remaining studies,^13^ (18% of those using surveys/interviews) described using an evaluation framework to inform their study, including tool development,^3^^,^^22^^,^^29^^,^^38^^,^^41^^,^^49^^,^^55^^,^^64^^,^^68^^,^^69^^,^^74^^,^^83^ of which 3^22^^,^^64^^,^^69^ referenced the same primary source^70^ – a scoping review whose goal was to develop a conference evaluation framework. Of the remaining 24 (32% of those using surveys/interviews), only two studies reported performing any pilot testing of their tool,^42^^,^^54^ with an additional three^29^^, ^^72^^, ^^80^ suggesting the work itself represented a pilot study for tool assessment.

### Content analysis: domains and subdomain identification

**Figure 3:**
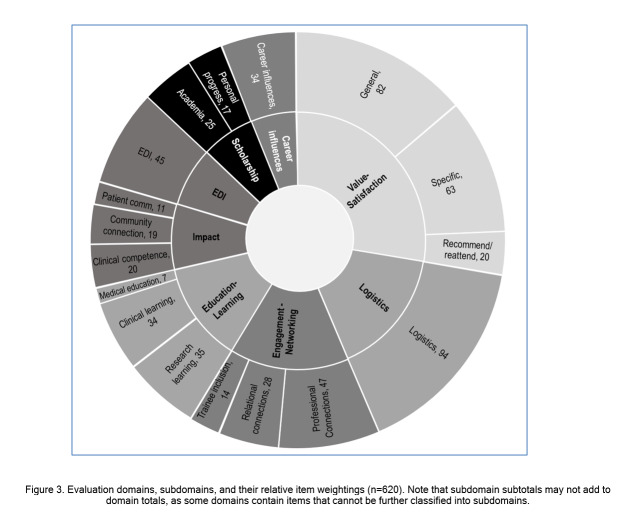
Evaluation domains, subdomains, and their relative item weightings (n=620). Note that subdomain subtotals may not add to domain totals, as some domains contain items that cannot be further classified into subdomains.

There were 620 individual items (evaluation questions or results obtained from surveys, interviews, and reported outcomes) identified and extracted from the studies, with a median of 6 items (IQR: 4-9) per study. As shown in Figure 2, there was a relatively stable average (median) number of items per study up to 2018, with the suggestion of a gradual increase from 2019 to 2022. Following content analysis, 8 major domains were identified (Figure 3), with the four a priori identified domains capturing only a minority of items (282, 45%). The four new domains identified during content analysis (value-satisfaction, logistics, equity-diversity-inclusivity (EDI), career influences) captured the majority of items (338, 55%).  Further item analysis identified subdomains within 5 of the domains, including all 4 of the a priori domains and value-satisfaction. Supplementary digital appendix 4 provides a more detailed description of the findings from content analysis including one ore more example item from each domain/subdomain. While no subdomains were identified for the logistics domain, analysis did recognize that the large number of items (n=94) evaluated a heterogenous group of characteristics such as location, timing, and various aspects of content delivery and organization, including a more recent focus on whether technology facilitated or hindered the delivery of other domains (e.g. education, networking). Consistent with the more recent focus on technology, 10 study tools (published 2020 or later) contained items specifically related to COVID-19 and ease of transition to virtual conferences, preferences for methods of information exchange, and/or success of social media promotion of the conference. Similarly, a clear time trend was evident for the EDI domain, with the 45 items all originating from 9 studies published in 2019 or later. The final and least featured domain was career influences which included items primarily related to whether the conference improved participants’ understanding of careers in an area, and/or increased motivation to pursue careers, professional development, or further training in the field. Thirty-one of the 34 items (91%) originated from studies evaluating conferences where students/trainees were included in the eligible population, with 27 (79%) being conferences held specifically for students/trainees.

## Discussion

This scoping review explored the published literature on CME conference evaluation with the goals of identifying validated instruments and relevant evaluation domains through content analysis. This work identified 83 studies originating from a range of medical fields, but no broadly applicable validated tools. While inspection of individual studies demonstrated that only a small minority described following recommended methodology for survey development (for instance, pilot testing), the extraction and analysis of over 600 individual items allowed for the identification of several domains and subdomains directly useful to future research in this area.

As expected, conference evaluation research was confirmed to be of widespread interest, spanning over two dozen medical fields and originating worldwide. While interest was widespread, the field of radiology and diagnostic imaging produced three times as many publications (n=10) as the average in all other fields (n=3). The higher volume may be linked to interventional radiology’s (IR) recent recognition as a primary specialty by the American Board of Medical Specialties in 2012 and need to recruit trainees into dedicated IR residencies.^59^ This is consistent with the observation that all 10 of the radiology studies reported on conferences specifically held for trainees, with several tracking conference attendance over time and student attraction to the program.

Analyzing study location identified that the majority of studies (72%) originated from North America. This proportion may be explained in part by study methodology factors (decision to only included English and French articles) or regional/cultural differences in approach to scholarship/publication or continuing medical education (CME); CME and professional development are highly regulated within Canada, the United States, and most Western European and Australasian countries, but vary more globally in terms of policies, infrastructure, and enforcement.^2^^, ^^102^^-^^104^ Additionally, the reality of ‘conference inequity’ – where most global health conferences (and therefore evaluations on these) are held in higher income countries^105^ – is also borne out here, as only 16% of studies came from outside of North America and Europe. While the publication rate appeared to plateau or only minimally grow between 2014-2019, at an average of seven per year, a spike was observed in 2020 to 11 publications. This spike timed with the onset of the COVID-19 pandemic, and as shown in Figure 2, these studies focused on assessing the impact of the necessary transition from in-person to virtual conferences. Although not implemented on a global scale prior to COVID-19, virtual conferences and webinars have been an important component of medical education prior to COVID-19 with proven success in knowledge transfer.

For instance, studies have shown improved standardized test scores following virtual lectures provided by first-world academic institutions to smaller hospitals in developing countries.^106^^,^^107^ While investigating the specific impact of virtual conferences is outside the scope of this research, seven of the 12 publications that evaluated virtual conferences reported attendee assessment as positive (i.e., the majority of participants reported equivalent or higher preference for the virtual format).^60^^,^^61^^, ^^68^^, ^^71^^, ^^79^^, ^^81^^, ^^94^ This was attributed to improved attendance, greater accessibility, and decreased environmental impact. Of the remaining publications, 3 of 12 did not specifically ask the participants about format preference, although respondents indicated that they had enjoyed the conference and were willing to continue meeting virtually;^42^^, ^^89^^,^^96^ 1 of 12 reported a majority preference for in-person meetings;^44^ and the final study reported approximately equal preference for in-person and hybrid/virtual.^82^

Some studies attempted to evaluate conference impact using objective metrics – such as examining links between attendance and performance on the American Board of Emergency Medicine In-Training Examination and U.S. Medical Licensing Examination,^39^ or distributing case study questionnaires to conference participants and non-participants to determine “whether the diagnostic and therapeutic choices of program participants were consistent with evidence-based guidelines”.^36^ Of the studies that used surveys, only one^97^ described using validation processes such as iterative revisions, factor analysis, and Cronbach’s alpha methods to assess internal consistency.^108^^,^^109^ While of clear value, this tool may have limited general applicability as it was specifically designed to measure a mobile device app’s impact on conference experience. In the absence of validated tools, some of the studies (n=13) sought out and described the consideration of previously published conference evaluation frameworks as part of tool development. Finally, only two studies^42^^, ^^54^ mentioned performing any tool refinement or pilot testing prior to implementation, widely considered essential steps in survey development.^110^^,^^111^ Despite the inability to formally assess the quality of each instrument included in our review, the lack of validity evidence supporting these instruments raises concerns about their methodological quality, as do other aspects of our general quality assessment (such as response rate reporting and clear sample population descriptors).

Our content analysis identified eight major evaluation domains. The traditional conference format is geared toward bringing individuals together, usually physically, for the purpose of shared learning – so the observed heavy weighting in these domains as well as in satisfaction and logistics supports the assumption that evaluation weighting parallels conference goals. This format often leads to new mentorship and professional development opportunities for those who attend, and there are well-documented challenges for those who do not or cannot attend.^105^^,^^112^^-^^115^ The four domains identified inductively addressed value-satisfaction, logistics, EDI, and career influences. Items assessing logistics and EDI were primarily found in more recent publications. More recent studies also tended to highlight concerns surrounding in-person conferences, such as the environmental impacts and attendance inequity. Both the identified studies and broader literature suggest factors like funding, inability to travel to conference location, limited speaking opportunities/representation, family/clinical commitments, and intrinsic feelings of belonging as barriers that disproportionately affect in-person conference attendance of women, minorities, and residents of lower income countries.^32^^,^^47^^,^^98^^,^^105^^,^^116^^-^^118^ Items addressing conference environmental impact and gender-related conference inequity were primarily found in studies^42^^,^^98^ published after 2019, indicating these to be emerging priorities within the scientific community. Virtual conferences have the potential to reduce environmental impacts and provide more equitable and convenient opportunities for networking, learning, and collaboration to all attendees.

Patients and caregivers are another group for whom inclusion has been a growing priority and seven studies within this review specifically included these individuals as stakeholders, potentially reflecting the growing importance their inclusion in conference planning and implementation has on preventing discrepancies between patient and health professional priorities.^38^^,^^41^^,^^63^^,^^69^ Patient and caregiver conference participation avenues varied, ranging from being the primary audience for improved education and involvement in medical and scientific discussions,^63^^,^^101^ to inclusion as planners and speakers to better incorporate their feedback into research, health care, and policy.^38^^,^^41^ This trend reflects a similar shift in broader health care and research toward patient inclusion.^119^^-^^121^ While this is demonstrably valuable and multiple organizations (e.g., Stanford Medicine X, Patients Included, European Patients Forum) have created charters for ideal methods of inclusion in conferences, further discussion within the medical community of how to meaningfully incorporate patients and caregivers from an EDI standpoint is warranted. The Stanford Framework for Patient Partnership, which was written to guide patient inclusion in CME conferences and “could also be used by prospective delegates to evaluate conferences they are contemplating attending,”^119^ suggests that accommodation, co-design, engagement, and education and mentorship should be guiding principles in meaningful inclusion.

This scoping review has strengths and limitations to be considered. One major strength is our application of a widely-accepted methodological framework^14^^,^^122^ for conducting scoping reviews. Through this approach we were able to thoroughly capture trends in CME conference evaluation research including the recent emergence of EDI, environmental concerns, logistics and patient/caregivers as important considerations. One major study limitation was our search restriction to MEDLINE®, deemed necessary given the absence of terms related to congress or conferences in other relevant databases. As recommended for difficult-to-search topics (in this case by the research topic and feasibility of a primary database search^123^), we used ancillary search methods and, in particular, citation searching.^124^ While limiting to a single electronic database may have reduced the number of eligible studies included we anticipate it to be without major effect as only 5 additional eligible citations were identified through citation searching that were not in our original MEDLINE® search, and these were conference abstracts, yielding little reliable evidence. A second potential limitation was the inclusion of only English and French articles, which may have reduced the number of conference evaluation studies outside of North American and Europe, and potentially limit generalizability to other regions and cultures.

## Conclusions

Through this scoping review we were able to map the published conference evaluation literature across many medical fields. This review did not identify a validated tool intended for conference evaluation, which suggests that organizers and research teams are developing their own instruments. While formal quality assessment was not performed, general quality assessment indicated that while study methodology was strong, tool development and recruitment techniques/reporting were weaker. This work confirmed the use of longstanding evaluation domains (e.g., education, networking) and revealed newer domains (e.g., EDI, found in studies published in 2019 or later) used in conference evaluations. The identification of domains, subdomains, and their relative weight may be useful to researchers seeking to evaluate future conferences, and to conference organizers to inform objectives, activities, and select indicators of success and impact. Additionally, by identifying widely-used domains (and subdomains) as well as trends in in-person vs virtual conference format, and by creating a database of sample items, this work helps set the stage for future projects aimed at developing more standardized evaluation instruments which can ultimately improve conference quality.

### Acknowledgements

The authors wish to acknowledge Lindsey Sikora, Head of Research Support (Health Sciences, Medicine, STEM) at the University of Ottawa, Ottawa ON for co-development of the search strategy.

### Conflicts of Interest

The authors declare they have no conflicts of interest.

## Supplementary material

Supplementary file 1 Appendix 1. Preferred reporting items for systematic reviews and meta-analyses extension for scoping reviews (PRISMA-ScR) checklist18

Supplementary file 2 Appendix 2. MEDLINE® search strategy

Supplementary file 3 Appendix 3. Variables extracted from studies in scoping review (insightScope instrument)

Supplementary file 4 Appendix 4. Sample items by evaluation domains and subdomains (n=620)

Supplementary file 5 Appendix 5. General quality assessment using elements common to quality assessment tools (AXIS125, QuADS126, MMAT127, MERSQI128, COSMIN129)
