# Supplementary material for: Measuring continuing medical education conference impact and attendee experience: a scoping review
Source: Int J Med Educ. 2024 Feb 29;15:15–33. doi: 10.5116/ijme.65cc.8c88 (PMC11285031; doi:10.5116/ijme.65cc.8c88)
Supplement: Supplementary file 2 — Appendix 2. MEDLINE® search strategy [file ijme-15-15-S2.pdf]

Appendix 2

MEDLINE® search strategy

Database	MEDLINE
Filename	Conferences – scoping review
	1. Congresses as topic/<br>2. *Congresses as topic/<br>3. Motivation/ or Achievement/ or Aspirations, psychological/ or Goals/ or Empowerment/ or Personal satisfaction/<br>4. Program Evaluation/<br>5. exp Education/ or exp Education, medical/<br>6. (engagement or impact or experience* or satisfaction or motivation* or evaluation* or effectiveness).ti,ab,kf<br>7. *Congresses as Topic/<br>8. 1 and (3 or 4 or 5 or 6)<br>9. limit 8 to (yr="2008 -Current" and (english or french))
