# Supplementary material for: Measuring continuing medical education conference impact and attendee experience: a scoping review
Source: Int J Med Educ. 2024 Feb 29;15:15–33. doi: 10.5116/ijme.65cc.8c88 (PMC11285031; doi:10.5116/ijme.65cc.8c88)
Supplement: Supplementary file 3 — Appendix 3. Variables extracted from studies in scoping review (insightScope instrument) [file ijme-15-15-S3.pdf]

Appendix 3

Variables extracted from studies in scoping review (insightScope instrument)

Demographics	1) Record ID<br>2) Article title<br>3) Date of data extraction<br>4) First author<br>5) Corresponding author<br>5b) Corresponding author contact information<br>6) Is there a second corresponding author? [Y/N]<br>6b) Second corresponding author contact information<br>7) Year of publication<br>8) Journal of publication<br>9) Country/region where work was performed [NORTH AMERICA, CENTRAL & SOUTH AMERICA, EUROPE, EAST ASIA, REST OF ASIA, AFRICA, AUSTRALIA/NZ, MIDDLE EAST, OTHER, NR/UNCLEAR]<br>9b) Country/region (other)<br>10) Do you have any other comments to make about study demographics for this paper? [Y/N]<br>10b) Comments
Study Information and Design	1) Record ID<br>2) Study type [SYSTEMATIC/SCOPING REVIEW, OPINION PIECE/COMMENTARY/EDITORIAL, NARRATIVE REVIEW, ORIGINAL RESEARCH, OTHER]<br>2b) Study type (other)<br>3) Type of original research [SURVEY/INTERVIEW/DELPHI, BIG DATA, INTERVENTIONAL TRIAL, OBSERVATIONAL TRIAL, TOOL VALIDATION]<br>4) Data collection methods [QUANTITATIVE, QUALITATIVE, MIXED METHODS, OTHER]<br>4b) Data collection (other)<br>5) Did the study population include or consider trainees? [Y/N/UNCLEAR]<br>6) Did the study population include or consider patients/caregivers? [Y/N/UNCLEAR]<br>7) Study objective was [NOT STATED, STATED BUT IN GENERAL OR NONSPECIFIC TERMS, STATED WITH ONE OR MORE OBJECTIVES CLEARLY DEFINED]<br>8) Primary study objectives<br>9) Secondary study objectives<br>10) Motivations of attendees evaluated [Y/N]<br>10b) If motivations for attending a conference were evaluated, please specify<br>11) Outcome measure evaluated is one or more of [IMPACT OF CONFERENCE ON STAKEHOLDERS, LEARNING/EDUCATIONAL ROLE AND VALUE OF THE CONFERENCE, ENGAGEMENT AND NETWORKING, SCHOLARSHIP, OTHER]<br>11b) Outcome measure (other)<br>12) Author's key findings<br>13) Gaps in literature<br>14) Do you have any other comments to make about study information and design for this paper? [Y/N]<br>14b) Comments
Conference Studied	1) Record ID<br>2) Conference(s) identified [Y/N]<br>2b) Conference name(s)<br>2c) Conference type [IN PERSON, VIRTUAL, NR]<br>3) Study describes conference activities [Y/N]<br>3b) Study considers impact of conference activities on some aspect of MILES (Motivations for attending, Impact on stakeholders, Learning/educational role and value, Engagement and networking, Scholarship) [Y/N]<br>4) Conference topic<br>5) Conference objectives<br>6) Conference duration reported [Y/N]<br>6b) Conference duration<br>7) Number of attendees reported [Y/N]<br>7b) Conference number of attendees<br>8) Number of participants in study reported [Y/N]<br>8b) Study number of participants

	9) Do you have any other comments to make about conference studied for this paper? [Y/N]<br>9b) Comments
Evaluation Tool/Framework	1) Record ID<br>2) Evaluation tool used [Y/N]<br>3) Evaluation tool validated [NONE, YES – FULLY VALIDATED TOOL, PARTIAL – SOME PILOTING AND TESTING OF TOOL DEVELOPED BY STUDY AUTHORS]<br>3b) Name of validated tool<br>3c) Year of validation<br>4) Reference for evaluation tool<br>5) Evaluation framework used [Y/N]<br>5b) References for evaluation framework<br>6) Evaluation tool provided [COMPLETE, PARTIAL, NO]<br>6b) Evaluation tool file<br>7) Method of recruitment [IN PERSON/AT CONFERENCE, VIRTUAL/ELECTRONIC, NOT REPORTED]<br>8) Measurement times [NOT STATED, BEFORE, BEGINNING, DURING, IMMEDIATELY AT END, POST]<br>8b) Duration of time for measurement (post)<br>9) Method of tool administration [ONLINE/VIRTUAL, AT CONFERENCE/IN PERSON, NR]<br>10) Potentially relevant citations [Y/N]<br>10b) Relevant citations<br>11) Do you have any other comments to make about evaluation tool/framework for this paper? [Y/N]<br>11b) Comments
