# Supplementary material for: Measuring continuing medical education conference impact and attendee experience: a scoping review
Source: Int J Med Educ. 2024 Feb 29;15:15–33. doi: 10.5116/ijme.65cc.8c88 (PMC11285031; doi:10.5116/ijme.65cc.8c88)
Supplement: Supplementary file 4 — Appendix 4. Sample items by evaluation domains and subdomains (n=620) [file ijme-15-15-S4.pdf]

Appendix 4

Sample items by evaluation domains and subdomains (n=620)

Domain	Subdomain	n (% of domain subtotal)*	Sample Item
Engagement-Networking	Professional Connections	47 (50.0)	I established a research collaboration with someone I met [at the conference].[27]
	Relational Connections	28 (29.8)	[How effective were you at] making contact or keeping in touch with others from [the conference]?[100]
	Trainee Inclusion	14 (14.9)	[How able were you to] engage in opportunities to identify appropriate mentors/mentees and attributes for future professional relationships?[88]
Education-Learning	Research Learning	35 (40.7)	I have a better understanding of educational research methodologies than prior to attending [the conference].[45]
	Clinical Learning	34 (39.5)	[Participants were asked to evaluate] surgical technique of live surgeon.[84]
	Medical Education Learning	7 (8.1)	[Did the conference affect] creation of a new or improved oral medicine training program?[29]
Impact	Clinical Competence	20 (35.1)	Did [the conference] help improve your neurosurgical practice?[81]
	Community Connection	19 (33.3)	[The conference] allowed me to think about practical applications of research data including policy work.[90]
	Patient Communication	11 (19.3)	Communication skills with patients will be more effective as a result of the conference.[21]
Scholarship	Academia	25 (55.6)	[Participants] presented research at a disciplinary society meeting because of attending [the conference].[32]
	Personal Progress	17 (37.8)	[The conference] helped increase [research] writing proficiency.[29]
Value-Satisfaction	General	82 (49.7)	[Participants were asked if they] found value in attending the conference.[99]
	Specific	63 (38.2)	How do you rate the academic level of the conference?[81]
	Recommend/ Reattend	20 (12.1)	[Participants were to indicate level of agreement with the following statements based on experience at the conference]: I encourage(d) others to attend [the conference], I plan to attend [the conference] in the future.[32]
Logistics		94 (100.0)	How would you rate your satisfaction with the virtual meeting platform for [the conference]?[61]
EDI		45 (100.0)	[Was] gender or implicit bias addressed at the conference or symposium which you attended?[98]
Career Influences		34 (100.0)	Are you interested in pursuing a career in diagnostic radiology?[59]

*Subdomain percentages may not add to 100% within all domains, as some domains contain items that could be further classified into subdomains.
