# Supplementary material for: Measuring continuing medical education conference impact and attendee experience: a scoping review
Source: Int J Med Educ. 2024 Feb 29;15:15–33. doi: 10.5116/ijme.65cc.8c88 (PMC11285031; doi:10.5116/ijme.65cc.8c88)
Supplement: Supplementary file 5 — Appendix 5. General quality assessment using elements common to quality assessment tools (AXIS125, QuADS126, MMAT127, MERSQI128, COSMIN129) [file ijme-15-15-S5.pdf]

Appendix 5

General quality assessment using elements common to quality assessment tools (AXIS125, QuADS126, MMAT127, MERSQI128, COSMIN129)

Quality indicator	Number of studies that addressed this (n=83), n (%)	Assessment tool reference
Study aims/objectives specifically OR generally defined	75 (90%)	AXIS #1, QuADS #2, MMAT S1
Study design provided (i.e., data collection method identifiable – e.g. 'mixed methods')	80 (96%)	AXIS #2, QuADS #4, MMAT #1.2/4.1/5.1, MERSQI #1
Target/reference population clearly defined (i.e. indication of whether patients AND trainees were included/not included)	57 (69%)	AXIS #5, QuADS #3
Strengths and limitations critically discussed (i.e., gaps in the literature noted)	46 (55%)	QuADS #13
For interview/survey studies (i.e. studies using a tool)	Number of studies that addressed this (n=74), n (%)	Assessment tool reference
Recruitment data provided (i.e., method of recruitment, timing of recruitment, AND method of tool administration provided)	55 (74%)	AXIS #6, QuADS #8-9
Tool rationale, format, content appropriate (i.e., tool provided)	22 (30%)*	QuADS #6-7, COSMIN checklist D
Tool developed using framework, piloted, OR validated (none of the studies did all of these)	17 (23%)	AXIS #9, QuADS #12, MERSQI #5-7, COSMIN checklist A-H
Response rate provided (i.e., sample size AND conference attendance provided)	42 (57%)	AXIS #13, MMAT #4.4, MERSQI #3

*An additional 17 (23%) provided portions of the tool
